# Australian occupational therapists' perspectives of consumers authentically contributing to student learning during practice placements: ‘It just makes sense!’ but ‘we need a process’

**DOI:** 10.1111/1440-1630.12853

**Published:** 2022-11-13

**Authors:** Thomas Bevitt, Stephen Isbel, Robert B. Pereira, Rachel Bacon

**Affiliations:** ^1^ Discipline of Occupational Therapy, Faculty of Health University of Canberra Bruce Australian Capital Territory Australia; ^2^ Hospital Admission Risk Program, Barwon Health Geelong Victoria Australia; ^3^ Discipline of Nutrition and Dietetics, Faculty of Health University of Canberra Bruce Australian Capital Territory Australia

**Keywords:** clinical education, consumer feedback, occupational therapy, placements, student

## Abstract

**Introduction:**

Collaborating with consumers in designing, delivering, and evaluating curricula is an ongoing initiative within occupational therapy tertiary courses in Australia. Within the Australian educational context, consumers are involved in on‐campus educational activities. Student occupational therapists must complete 1000 hours of practice placements as part of their education. To date, no research has explored how consumers could contribute to student occupational therapists' learning during practice placements. This study aimed to explore Australian occupational therapists' perceptions of consumers providing feedback to students during practice placements.

**Methods:**

A qualitative descriptive approach was adopted to engage with the diversity of practice contexts and gain a rich dataset from the occupational therapy profession. A qualitative questionnaire was developed and distributed using snowballing techniques. The questionnaire asked recipients to reflect on the risks, challenges, and benefits of consumers providing feedback to student occupational therapists from all stakeholders' perspectives. Demographic data were collated, and reflexive thematic analysis was used to construct themes.

**Findings:**

Responses were received from 81 participants. Most respondents identified as experienced occupational therapists from metropolitan locations across Australia. Reflective thematic analysis was used to construct three themes: Personal capability of consumers and students will enable, inhibit, and be developed by engaging in a feedback process; an educator‐controlled process to ensure safety for all stakeholders is required for time‐poor practice contexts; and us versus them: Shifting control to consumers can disempower practice educators.

**Conclusion:**

Engaging with consumers throughout all aspects of student occupational therapists' educational programme is required, including practice placements. New educational initiatives need to consider all stakeholders' concerns to ensure that authentic contribution from consumers is made within the various practice contexts. A co‐design approach that involves all stakeholders to develop a feedback process may result in high‐quality learning experiences that assist students to become safer, consumer‐centred health professionals.

**Key Points for Occupational Therapy**

- For students to become safer, consumer‐centred professionals, consumers need to authentically contribute to learning during placements.
- A consumer feedback process needs to be developed to ensure safe, high‐quality learning experiences.
- Training and education need to be provided to consumers, practice educators, and students to address assumptions and concerns.

## 1 INTRODUCTION

National and international occupational therapy course accreditation guidelines state that educational programmes must ensure that a consumer perspective is integrated within the design, delivery, and evaluation of student occupational therapists' learning (Occupational Therapy Council of Australia, 2018; World Federation of Occupational Therapy, 2016). ‘Consumer’ describes any person or carer who uses, has used, or may use any health or health‐related service (Australian Commission on Safety and Quality in Health Care, 2021). In Australian occupational therapy education programmes, consumers have contributed to on‐campus education via curriculum development, delivering and assessing course content, as well as performing roles of instructors, and ‘standardised’ patients within simulations (Scanlan et al., 2020; Soon et al., 2020). Predominantly, research has focused on mental health consumer engagement to assist with de‐stigmatising mental health and teaching recovery‐based practice (Arblaster et al., 2018; Scanlan et al., 2020). Recent findings into consumers' experiences of the National Disability Insurance Scheme have also been suggested to guide curriculum development (Barclay et al., 2020). Consumers articulated the importance of including people with lived experience of disability to teach the students to recognise that the person with a disability is the expert on their own needs and to work with the person and not treat a ‘disability’ or ‘condition’ (Barclay et al., 2020). Consumers want educational curricula to focus on person‐centred practice, highlighting the importance and impact a high‐quality therapeutic relationship via effective active listening and rapport has on productive outcomes for the consumer (Barclay et al., 2020).

Occupational therapy international accreditation standards require students to complete 1000 hours of practice education (World Federation of Occupational Therapy, 2016). Practice placements, hereafter referred to as placements, are a period where students interpret and apply occupational science and therapy theories in a professional context with consumers (World Federation of Occupational Therapy, 2016). Placements are a signature pedagogy of occupational therapy education (Penman et al., 2021). Signature pedagogies are fundamental ways the profession teaches future professionals how to think, perform, and act with integrity to develop professionally valued habits of head, mind, and heart (Penman et al., 2021; Shulman, 2005). Signature pedagogies often require the student to perform in public, whether in a classroom or during a placement (Shulman, 2005). Signature pedagogies have been identified for medical and nursing education, and descriptions are developing for occupational therapy education (Penman et al., 2021). Three inter‐related signature pedagogies have been identified in occupational therapy placements: Learning is relational, affective, and highly contextualised (Schaber et al., 2012). Penman et al. (2021) have described two distinctive features of feedback as a signature pedagogy during occupational therapy placements: creating a comfortable learning environment through feedback and achieving feedback for learning.

Feedback during placements is ‘a process whereby a learner obtains information about their work in order to appreciate the similarities and difference between the appropriate standards for any given work, and the qualities of the work itself, in order to generate improved work’ (Boud & Molloy, 2013, p. 6). Feedback as a construct for learning is now understood to be an active process between students and educators. The student must play a role in seeking, making sense of, and translating new knowledge into demonstrated changes in practice performance (Molloy et al., 2019). Students' underlying skills and abilities to actively engage, make sense of, and apply learnings from feedback have been described as feedback literacy (Molloy et al., 2019). In Australia, practice educators (also known as supervisor or preceptor) provide most feedback to the student across various points throughout occupational therapy placements (Penman et al., 2021). Research has investigated feedback processes provided by peers on peer‐assisted learning placements or near peers (Penman et al., 2021).

In Australian placement settings, feedback and student performance observations are collated and summarised by the practice educator into a formal assessment called the Student Practice Evaluation Form‐Revised 2 (SPEF‐R2). The SPEF‐R2 has a formative and summative evaluation (Caine et al., 2021). The assessment tool was recently updated to incorporate the new professional competency standards developed by the Occupational Therapy Board of Australia (Caine et al., 2021). The SPEF‐R2 instructions provide guidelines for supervisors on how to manage feedback and an evaluation process between qualified occupational therapists and students. The SPEF‐R2 suggests a role for consumer feedback within the assessment tool; however, no guidance is provided for supervisors on how to manage consumer feedback.

The most recent international systematic mapping of practice education research did not identify any research exploring consumers providing feedback to students and their impact on learning during occupational therapy placements (Roberts et al., 2015). Research investigating consumers providing feedback to students during placements is predominately by nursing and medicine programmes in Europe and the United States of America (Finch et al., 2018; Khalife et al., 2022; Suikkala et al., 2018). ‘Consumer feedback’ is described as a deliberate and specifically designed educational technique for learning (Finch et al., 2018; Suikkala et al., 2018). The predominant form of gathering consumer feedback for student learning is via purpose‐developed templates using Likert scales and open‐ended questions (Finch et al., 2018; Freeman et al., 2019; Masters & Forrest, 2010). Medical and counselling education programmes have used standardised feedback tools for consumers to comment on students' communication skills (Bogetz et al., 2018; Mahoney et al., 2019). Nursing education programmes in Scotland have developed a national placement assessment tool that incorporates consumer feedback (Masters & Forrest, 2010). Consumers have also contributed to formal supervision and feedback sessions with nursing students completing mental health placements (Debyser et al., 2011).

Consumer feedback processes have focused on appraising professional behaviours and communication skills (Bogetz et al., 2018; Freeman et al., 2019). Research has found that consumer feedback assists students with the development and demonstration of communication skills, empathy, and confidence (Bogetz et al., 2018; Masters & Forrest, 2010). Internationally, research has found that health professional practice educators have initial apprehensions about approaching consumers for feedback on student performance (Duygulu & Abaan, 2013; Haycock‐Stuart et al., 2016; Speers & Lathlean, 2015). Concerns focused on risks and challenges and questioned if the risks outweighed the benefits for all stakeholders (Freeman et al., 2019; Haycock‐Stuart et al., 2016; McComb et al., 2019; McMahon‐Parkes et al., 2016). Family therapy practice educators' attitudes towards consumers giving students feedback strongly influenced students' engagement rates and successful learning outcomes (McComb et al., 2019).

When innovating existing educational practices, it is important to develop an understanding of each stakeholders' perspectives and considerations of what they think will contribute to a successful system (Haycock‐Stuart et al., 2016). Although there is no specific research on the perspectives of occupational therapy consumers giving feedback to occupational therapy students, research does exist on consumers giving feedback within other health fields (Finch et al., 2018; Masters & Forrest, 2010; Suikkala et al., 2018). The viewpoints of occupational therapists on consumer feedback during placements have not been examined. Therefore, this paper sought to answer the research question: What are Australian occupational therapists' perspectives of consumers actively contributing feedback to student occupational therapists during placements?

## 2 METHODS

### 2.1 Design

This research adopted a qualitative research approach, as it sought to develop a rich description of a phenomenon where little is known and enable researchers to learn from the descriptions to influence future interventions (Bradshaw et al., 2017; Sandelowski, 2000, 2010; Stanley, 2015). The research question acknowledges that understanding phenomena is subjective and varies from person to person (Bradshaw et al., 2017). Qualitative descriptive methods result in rich data when target participant perspectives are from diverse populations from large geographical areas and when researchers and participants have limited time and resources (Bradshaw et al., 2017; Sandelowski, 2000, 2010; Stanley, 2015). Ethical approval was granted by the University of Canberra (project ID: 218) before participant recruitment occurred.

### 2.2 Participants

This research sought the perspectives of Australian Health Practitioner Regulation Agency (AHPRA)‐registered occupational therapists with experience supervising student occupational therapists. It is unknown how many AHPRA‐registered occupational therapists supervise students. Therefore, purposive sampling using snowballing methods was implemented (Streeton et al., 2004; Wilson, 2016). Participants self‐identified as AHPRA‐registered occupational therapists and practice educators to participate in the study. A questionnaire was distributed from September 2018 to December 2018. The questionnaire was distributed via the university's professional contact list, the Australian and New Zealand Occupational Therapy Fieldwork Academics (ANZOTFA), and the Australian and New Zealand Council of Occupational Therapy Educators (ANZCOTE) email distribution list. The email requested recipients to forward the email to known contacts. A social media campaign was implemented via Facebook, Twitter, LinkedIn, and the Occupational Therapy Australia web page. A paid advertisement was run via Facebook. An email reminder and social media alert were sent 1 month before the questionnaire closed.

### 2.3 Data collection

A qualitative questionnaire was constructed using Qualtrics™ to collect data (Qualtrics, 2022). Qualitative questionnaires offer a ‘wide‐angle lens’ to capture diverse perspectives and sense‐making when researching an unexplored area (Braun et al., 2021). Online qualitative questionnaires collate a rich dataset via open‐ended responses to a list of standardised questions. Qualitative questionnaires have been noted to be unobtrusive and less burdensome and reduce power imbalances for participants by removing the need to travel or host researchers and by being able to be completed alone at a time and location convenient for the participant (Braun et al., 2021). Demographic questions were designed to describe the participants, their practice context, work location, professional practice areas, and level of expertise in supervising student occupational therapists. The questionnaire used AHPRA registration categories for professional demographics, specific descriptions for the level of experience in supervision, and the Australian Geography Standard descriptors to define geographical work location (Australian Bureau of Statistics, 2018). A series of open‐ended questions were designed based on a comprehensive literature review conducted by the authors. The free text questions required participants to reflect on their current work context and consider the risk, challenges, and benefits of requesting feedback from consumers for student learning. The questionnaire requested consideration from the student, consumer, practice educator, and practice context perspectives. A final question asked what resources or support the practice educators would require to gather consumer feedback. At the time of data gathering, the SPEF‐R was the student assessment used during placements. The SPEF‐R2 was being developed and not in use. A copy of the questionnaire has been provided in the Supporting Information.

Five AHPRA‐registered occupational therapists and three non‐occupational therapists piloted the questionnaire for content validity, readability, and usability before distribution (Braun et al., 2021). Following feedback, the questions' wording was modified, and the questions were reordered for flow. Technical modification to the Qualtrics™ forms was made to assist with readability for handheld devices. Examples of the changes made are included in the Supporting Information. Pre‐testing also helped estimate completion time. The final survey consisted of 20 questions: seven demographic questions, three closed‐ended questions, and 10 open‐ended questions. Participants were provided an explanatory note before indicating consent by clicking ‘next’ to participate.

### 2.4 Data analysis

Usable questionnaires were defined as having responded to all open‐ended questions, and 80% of the demographic information to ensure the population could be described. Demographic data were collated. When no respondent selected an option in the questionnaire tool, it was excluded from the results table. Qualitative data were exported into Microsoft Excel in question order to retain the context of the response. The data were viewed as a whole. Reflexive thematic analysis was completed using Braun and Clarke's (2022) six stages in an iterative way. The first author worked with the whole dataset. SI, RP, and RB were provided with a third of the responses to all questions. Authors initially worked individually to develop their own codes and interpretations of the data.

1. Data familiarisation and writing familiarisation notes

Authors read through their sections of data at least twice, journaling thoughts generated and noting assumptions and perspectives while reading. Notes for potential codes were listed separately.

- Systematic data coding

Authors systematically reviewed their sections of data, giving full and equal attention to each item. Codes were created by identifying a feature of the data that appeared interesting with a note explaining and exploring the feature. Codes were made for as many potential themes as possible. All data were coded. Data were coded with as many different codes as required.

- Generating initial themes from coded and collated data

Authors reflected on their lists of codes and used tables to organise and generate candidate themes. The authors then met to discuss their individual analysis processes, codes, and candidate themes. During the deliberations, the authors provided reasoning about why and how they interpreted the data and assumptions they were bringing from their lived experiences. The first author maintained notes of the discussions and was provided copies of the other authors' codes and candidate themes. The first author then reread, reviewed, and continued developing codes and candidate themes for all data. No candidate themes were discarded at this stage.

- Developing and reviewing themes

The first author examined candidate themes for fit with the evident meaning of the whole dataset and coded any additional data that may have been missed. Specific quotes that represented the theme were noted.

- Refining, defining, and naming themes

The first author presented named themes with descriptions and supporting quotes to all authors for deliberation. Specific terms and descriptions of the themes were refined following deliberation to ensure the scope, and content of the theme was representative of the whole dataset. The first author reread the whole dataset to determine if the theme map was encompassing the whole dataset.

- Writing the report

The first author completed an initial draft of the report, with all authors contributing to the iterations of the manuscript. As revisions developed, the first author kept referring to the original dataset to ensure amendments to theme terminology, and supporting quotes were reflective of the whole dataset. A sample of the reflective thematic analysis is provided in the Supporting Information.

### 2.5 Research team, reflexivity, and positioning

This research was completed by three occupational therapists and one dietitian from various academic and professional practice roles. TB, SI, and RB are full‐time academics with diverse professional experience in occupational therapy and dietetics. These authors currently work with students and practice educators during placement experiences. Their role is to support student learning, practice educator teaching during placements, and make a fair and reasonable judgement about student performance based on presented evidence. RP is a full‐time practice professional and researcher. He works with students during placements and part‐time academic teaching.

The first authors maintained an electronic audit trail of analytic decisions by saving multiple versions of coding and candidate themes as they were developed. A handwritten journal capturing additional thoughts and rationale was also maintained throughout the whole analysis process and research meeting discussions. The analysis process occurred over 6 months, with meetings and discussions planned to allow space between discussions to encourage further reflexivity.

## 3 FINDINGS

Eighty‐one usable responses were gathered. Seven responses did not meet the completion requirements. Of the seven responses, little to no data were recorded in attempts. The demographic data are presented in Table 1. Respondents were from all states and territories in Australia except for the Northern Territory. Most respondents were from a major city. Respondents were from various practice areas, with acute, disability, mental health, paediatrics, and rehabilitation creating 62% of respondents. Most respondents indicated that they were proficient or expert student supervisors.

**TABLE 1:** Demographic characteristics

Demographic characteristic	*n*	%
State		
Australian Capital Territory	18	22
New South Wales	10	12
Queensland	26	32
South Australia	1	1
Tasmania	2	3
Victoria	16	20
Western Australia	4	5
No response	4	5
Work location		
Major city	54	67
Inner regional	19	24
Outer regional	4	5
No response	4	5
Primary scope of practice		
Academic/clinical education	10	12
Acute care/surgical	10	12
Aged care	4	5
Community	7	9
Disability	12	15
Driving	1	1
Hand therapy	2	3
Metal health	8	10
Paediatrics	12	15
Rehabilitation	8	10
Other	3	4
No response	4	5
Years of experience		
0–5	14	17
6–10	9	11
11–15	13	16
16–20	11	14
21–25	13	16
26–30	9	11
31–35	3	4
36–40	4	5
41–45	1	1
No response	4	5
Experience supervising		
Novice	10	12
Advanced beginner	2	3
Competent	11	14
Proficient	26	32
Expert	26	32
No response	6	7

### 3.1 Themes

Three themes were generated through the reflective thematic analysis: Personal capability of consumers and students will enable, inhibit, and be developed by engaging in a feedback process; an educator‐controlled process is required for time‐poor practice contexts to ensure safety for all stakeholders; and us versus them: Shifting control to consumers can disempower practice educators.

#### 3.1.1 Theme 1: Personal capability of consumers and students will enable, inhibit, and be developed by engaging in a feedback process

The personal capability of the consumer and the student was the most prominent theme. Capability was seen at both a semantic and latent level and was particularly evident with questions that focused on perceived risk. The respondents described capability as the skills, knowledge, and behaviours of the consumer and student that would enable safe, high‐quality, and low‐risk learning experiences. Respondents discussed skills, knowledge, and behaviours that consumers and students may lack that would prevent a successful interaction, ‘Students may not have the experience to interpret or tease out meaning in a non‐professionals delivery of feedback’ (R5). Few responses identified behaviours that need to be present to afford a successful feedback engagement.

Respondents also identified potential for personal development for the consumer and professional development for the student via a feedback process. When discussing an absence of skills, knowledge, or behaviours for both consumers and students, respondents often used words that suggested that they were drawing on past experiences with consumers and students and anticipating similar responses to occur when engaging in a feedback process. For example, there was an expectation that some students would respond in a similarly defensive way to constructive consumer feedback as to a practice educator's critique, ‘[feedback] Can be confronting, and most students don't take criticism well’ (R12), and that consumers may be harsh in their feedback delivery, ‘Consumers [are] not able to provide constructive criticism which students may take harshly or the wrong way’ (R3).

The capability of ‘knowing’ formed an element of this theme. Respondents queried consumers' ability to know what high‐quality practice ‘looked like’ and how to provide insightful, constructive, and safe feedback for themselves and the students.

They [consumers] would not know what level the student is at and therefore could not give a fair feedback on the student. Their experience with the student is limited to professionalism, communication etc but not in the context of what year level or what expectation they might have of the student. (R7)

Students were perceived as not knowing how to accurately and appropriately receive, process, and apply critical feedback. ‘It may be problematic to provide the raw consumer feedback to the student and may need the student supervisor to assist in interpreting the feedback’ (R52).

Respondents described situations that may support the identified gaps in capability, such as the length and depth of the relationship with the student. Respondents determined that a longer relationship between consumer and student may assist the consumer in understanding the expected professional standards and provide more accurate and high‐quality feedback. ‘The consumer needs to have already built a good rapport with the student to enable reliable feedback ‐ so might be helpful on longer placements’ (R11). It was also suggested that training for the student and consumer could be an opportunity to address gaps in capabilities.

Consumer and student capability was thought to be enhanced if they engaged in a feedback process. Some participants suggested that consumers may be empowered when giving feedback to students, developing their skills in being more confident and engaged in their health care. The increased consumer empowerment was not always viewed as a benefit. Some responses suggested that placement sites may be concerned that empowered consumers might increase their critique of the service.

It will be empowering for the consumer. They will feel like active participants in their care. It offers a new role for them to engage in. It will support them to develop their skills in providing feedback to health professionals, which may extend to others of their life, i.e. advocating for themselves. (R33)

The setting may be concerned that if consumers are empowered to give feedback to students, this might result in more feedback being provided about the service/setting/other health professions. (R25)

Concurrently, respondents identified that involving consumers more directly could assist the student in developing high‐quality practice skills. Respondents consistently linked constructive learning to positive consumer engagement experiences. Only one respondent suggested that critical feedback had the potential to be a constructive powerful learning experience.

The student can gain greater confidence in how they are working with clients from their perspective. The feedback is real and authentic, and is direct. The feedback received will aid in the [student] occupational therapist being a more consumer‐focused clinician as direct feedback from people who they have served is quite powerful. (R12)

#### 3.1.2 Theme 2: An educator‐controlled process is required for time‐poor practice contexts to ensure safety for all stakeholders

Respondents saw value in consumer feedback for student learning and expressed a need for a clear and pragmatic process to be developed. When discussing process elements, responses used terms that indicated that the practice educator would be in control of the process to ensure safety for all stakeholders. Respondents wanted ‘[Potentially] a university form that would legitimise the process (R1)’ and justify any additional time it may take to safely identify consumers, gather feedback, and discuss with the students. The SPEF‐R is featured prominently throughout this theme. Respondents identified that as the SPEF‐R did not specifically outline a process, they did not realise that consumer feedback was an option. ‘It [the SPEF‐R] doesn't prompt me to request feedback from consumers. I find this as quite limiting as their feedback would be vital for student growth’ (R25).

When discussing the pragmatic requirements of a process, most responses raised concerns about practice educators' availability of time to manage the process, time to identify consumers, time to train consumers, and time to discuss the feedback with students, especially when consumer feedback was perceived as non‐constructive. Some respondents felt that time spent implementing a new process had the potential to reduce ‘actual’ therapy time with consumers and learning time with health professionals. One recipient mentioned that consumer feedback might assist with reducing the amount of time the practice educator would spend giving feedback.

If nonconstructive feedback is provided then the practice educator needs to be able to manage this to help rebuild confidence, which takes time in a time‐poor environment. (R21)

Balancing clinical practice needs and student supervision needs with consumer feedback ‐ if not planned appropriately, could lead to reduced clinical time (e.g., reduced amount of rehab practice). (R8)

Responses that spoke to time indicated that the practice educators felt responsible for managing the whole process to ensure the safety of all stakeholders. To assist with managing risk for all stakeholders, respondents spoke of facilitating the feedback process, *‘*hearing real life consumer feedback, very powerful, and should be facilitated at all times’ (R63), and ‘also, no real opportunity to engage with the consumer without the student present’ (R41). Respondents assumed that they would identify which consumer should be asked, coordinating, sourcing, filtering, and delivering the consumer feedback to students. Few respondents suggested that the feedback would be face‐to‐face and direct between students and consumers.

The PE [practice educator] would need to choose suitable consumers to provide, which may be difficult in a setting where the consumer is only seen once or twice (not much time to get to know the consumer and how suitable they would be in providing feedback). (R33)

#### 3.1.3 Theme 3: Us versus them: Shifting control to consumers can disempower practice educators

The final theme explored a complex perception of a power shift from the practice educator to the consumer in the student education process. There was a perception that directly including consumers in the student's education may disrupt the power relationship between the practice educator and the student. The ‘power’ concept between supervisor and student was noted within all questions on risks, challenges, and benefits. Some respondents explicitly used the word ‘power’, whereas others discussed how formally engaging consumers in student learning may create conflict and question the practice educator perspective—an ‘us and them’—the professional perspective versus the consumer perspective. ‘If the consumer provides positive feedback and the educator has a different perspective on how the services were delivered it can be difficult to provide conflicting feedback’ (R17). ‘[with consumer feedback] The practice educator may feel their “power” in the student/relationship is diminished or shared’ (R5). A change in power was also identified as favourable, with some respondents encouraging the shift.

Power was discussed specifically regarding the appraisal of student performance. Respondents reported concern that consumer feedback may contradict the practice educator's feedback. Respondents' examples within the responses consistently indicated that the consumer feedback perspective was ‘wrong’ and the practice educator's interpretation of student performance was ‘right’. There was a related concern that any conflict in the opinion of student performance would be challenging for the practice educator to resolve, requiring the practice educator to justify why their perspective was ‘right’. Some data suggested that respondents perceived the consumer perspective as not as important or as valuable as the practice educator teaching.

Feedback provided by consumer may be positive while feedback from supervisor may be negative, students who lack insight around their own performance and the limitations of obtaining consumer feedback may then use this to justify their view of their performance. (R26)

## 4 DISCUSSION

This study is the first known to investigate Australian occupational therapists' perceptions of consumers contributing feedback to student occupational therapists' learning during placements. This research found that practice educators perceived consumer feedback as a potentially powerful and empowering opportunity for both consumer and student occupational therapists but requires a comprehensive process to ensure a safe interaction for all stakeholders. The overall interpretation of the findings focused on a central concept of ‘process to ensure safety’. This study found that respondents perceived that a well‐designed process would address gaps in student and consumer capabilities, reduce risk, increase safety, and address concerns surrounding the practice educator's availability of time to provide feedback. Integral to the concept of ‘process to ensure safety’ for educators was the notion of control of the feedback process between student, consumer, and educator. Without a straightforward feedback process, educators feared that the power relationship between the student and the practice educator could compromise learning for the student and safety for the consumer.

This research confirms findings in similar research that the safety of all stakeholders is a primary consideration when developing a consumer feedback process (Balasa et al., 2021; Debyser et al., 2011; Stickley et al., 2011). When creating a new educational initiative, designers are encouraged to examine the assumptions used to justify concerns around ‘safety’ and ‘risk’ (Coelho et al., 2018). This research found that practice educators perceived that students might not be capable of seeking and processing critical consumer feedback based on their experiences of how students responded to practice educator feedback. There are two assumptions within this finding: First, consumers will deliver harmful feedback to students, and second, students will process consumer feedback similarly to practice educator feedback. The perception that students will process consumer feedback similarly to practice educator feedback is contrary to existing research that has shown that nursing, medical, and counselling students perceive and process consumer feedback differently to practice educator feedback (Speers & Lathlean, 2015). Students can perceive consumer feedback as authentic, legitimate, non‐threatening, and non‐judgemental, particularly if the feedback provider is not an assessor responsible for the students progressing in their studies (Finch et al., 2018; Speers & Lathlean, 2015). In some instances, consumer feedback with processing support from trusted mentors is more influential on students learning than lectures, practice educator feedback, and standardised placement assessment forms (Stickley et al., 2010). In occupational therapy, students completing simulated learning experiences have demonstrated effective engagement in a consumer feedback process (Springfield et al., 2018; Walls et al., 2019). However, it is acknowledged that simulation provides a structured and more controlled environment, and it is unknown how occupational therapy students will respond to consumer feedback in placement settings (Walls et al., 2019).

Our research also found that practice educators identified a concern with consumers' ability to safely engage in a feedback process. This finding is also contrary to existing research. Consumers have demonstrated feedback literacy skills by identifying themselves as ‘co‐educators’ in student development. Consumers are conscious of the impact their feedback has on student learning and provide predominantly positive feedback (Coelho et al., 2018; Suikkala et al., 2018). However, consumers identify that it is their responsibility to provide a constructive critique of the student performance to assist the students in their learning and to protect the public (Coelho et al., 2018). Consumers report confidence to provide a critique of student performance when they are informed of the requirements of the feedback, their input is valued, and the practice context provides support (Balasa et al., 2021; Carty et al., 2020; Coelho et al., 2018; Suikkala et al., 2018). Critical consumer feedback has also been viewed constructively by the student and led to advances in student learning and professional skill demonstration (Finch et al., 2018; Suikkala et al., 2018). As supported by these research findings, a consumer feedback process needs to create an engaging environment where students and consumers feel confident in providing feedback to students.

This study found that practice educators were concerned that consumer feedback could be counterproductive if not tightly controlled. Educators in our study wanted to ‘control’ the feedback process out of a genuine concern for each stakeholder's safety in the absence of an explicit feedback process. Nursing and medical education research provide similar debates regarding control and safety (Balasa et al., 2021; Björklund et al., 2021). Concerns of stakeholder safety are often addressed by developing feedback processes that create anonymous interactions between consumer and student with feedback being filtered and delivered to the student by the practice educator (Balasa et al., 2021; Coelho et al., 2018; Debyser et al., 2011; Stickley et al., 2010; Suikkala et al., 2018). Although anonymous and non‐direct approaches to feedback can address potential safety concerns, there are several counterarguments developing in recent research.

An alternative viewpoint to the concepts of control based upon some of the findings in this study is that educators should continue to reflect on the nature of the power and control between consumer, student, and practice educator in the learning environment. The role of the hidden curricula within our health and education systems has been identified as a contributor to a paternalistic culture under the guise of safety and risk (Bleakley & Bligh, 2008; Coelho et al., 2018). Our findings challenge occupational therapists to reflect on some of the assumptions around risk, safety, and power when using feedback from consumers in a practice education context.

Direct consumer feedback may assist with preparing the students for employment, where consumer feedback occurs on the consumers' terms. Direct consumer feedback may enable the student to develop and demonstrate professional behaviour and communication competencies (habits of hand, heart, and mind) that genuinely and authentically occur during interactions with consumers (Bogetz et al., 2018; Coelho et al., 2018). The opportunity to develop these skills is less likely to occur when anonymous and non‐direct forms of feedback are used within supervision sessions (Bogetz et al., 2018; Coelho et al., 2018). By creating a consumer feedback process as closely aligned with the practice context processes, students and consumers can develop and demonstrate a greater depth of skills (Billett, 2019).

For feedback to be effective for learning, students must be active participants in the process (Molloy et al., 2019). Control in anonymous and non‐direct feedback approaches often lies with the university or practice educator, and the student can become a passive recipient of feedback (Bogetz et al., 2018; Molloy et al., 2019). Direct consumer feedback would require the student to be an active participant in the feedback process. The student would be required to reflect and consider, in collaboration with the practice educator and consumer, what professional competencies they would like to seek feedback on. As active participants in the feedback process, the students would be able to consider their current learning needs and develop strategies to gather feedback with the consumer.

Direct consumer feedback allows the student and consumer to openly discuss the feedback provided. Anonymous and non‐direct consumer feedback approaches have been found to elicit positive and broad generalised feedback that students find difficult to apply for learning (Bogetz et al., 2018). Direct consumer feedback may provide the opportunity for the learner to seek clarification and understanding of what they did well, as well as discuss with the consumer strategies on how they could improve (Bogetz et al., 2018; Coelho et al., 2018). A consumer feedback process needs to authentically transition the consumer from a passive role in the educational relationship with the student to a co‐educator and the practice educator providing expert support for learning (Bleakley & Bligh, 2008; Coelho et al., 2018).

## 5 LIMITATIONS

This study aimed to gather perspectives from the breadth of occupational therapy professionals and has been completed by academics. No consumer involvement in the design and analysis of this research provides a limited interpretation of the research question. As outlined in the research design, this research did not seek to be representative. The respondents were of a relatively homogenous group, mainly experienced occupational therapy practice educators from metropolitan practice settings, with low participant numbers from two states and none from the Northern Territory. Further research is required to capture the perspectives of practice educators with less supervision experience and professionals working in rural practice contexts. Further research could explore if these professionals shared similar perspectives on consumer and student capabilities, pressure from practice context, and concerns with sharing power. Questions prompted respondents to reflect on the SPEF‐R, not SPEF‐R2. The SPEF‐R2 has an increased focus on reflective practice and a suggestion that consumer feedback could be used. However, no specific consumer feedback process is outlined in the SPEF‐R2. Further research could investigate if the changes in the SPEF‐R2 impact how practice educators engage with consumers for student learning.

## 6 IMPLICATIONS FOR PRACTICE

A feedback process needs to be developed that actively and authentically engages consumers in student occupational therapists' education during placements. The development phase must consider consumer rights and responsibilities, student learning, and practice context. A feedback process needs to allow for the safe sharing of control between all stakeholders to enable authentic consumer contributions to learning, to assist students in becoming safer, consumer‐centred health professionals. Training and education requirements for each stakeholder must be considered to address assumptions and concerns around the safety and risk of all stakeholders.

## 7 RECOMMENDATIONS FOR FUTURE RESEARCH

Further research is required regarding all stakeholders' perspectives on how consumers could authentically contribute to student occupational therapists' education during placements.

The tension between the practice educators wanting to filter and control consumer feedback and the need for the students to receive direct authentic feedback is intriguing and requires further exploration.

A co‐design approach to developing, implementing, and evaluating a consumer feedback process needs to be completed to determine the effectiveness of the process for all stakeholders and the design team. Further research is required to investigate the experiences of the process from all stakeholders' perspectives to identify enablers and barriers to the process. Other areas to investigate include the signature pedagogy of consumer feedback for student learning, the impact engaging in the process has on specific competence development for the student, and any impact on the consumer and practice educator.

## CONFLICTS OF INTEREST

Stephen Isbel is a member of the Australian Occupational Therapy Journal Editorial Board.

## AUTHOR CONTRIBUTIONS

All authors contributed to the study design and development of the data collection tool and analysis. The first draft of the manuscript was written by Thomas Bevitt as part of a PhD. All authors developed all versions of the manuscript.

## Supporting information


**Data S1.** Supporting Information

**Data S2.** Supporting Information

## Data Availability

Research data are not shared.
