# Supplementary material for: Australian occupational therapists' perspectives of consumers authentically contributing to student learning during practice placements: ‘It just makes sense!’ but ‘we need a process’
Source: Aust Occup Ther J. 2022 Nov 13;69(6):753–65. doi: 10.1111/1440-1630.12853 (PMC10098716; doi:10.1111/1440-1630.12853)
Supplement: Supplementary file 1 — Data S1. Supporting Information [file AOT-69-753-s001.docx]

Questionnaire

During practice education placements, occupational therapy students receive formal feedback primarily from the supervisor, professional colleagues and student peers. To date, formal feedback from consumers who receive direct service and interactions from occupational therapy students in Australia has not been documented.

Are you currently an occupational therapist registered with Australian Health Practitioner Registration Agency (AHPRA)?

- Yes – continue with survey
- No – thank you for your time

When did you graduate from your occupational therapy course?

1. Prior to 1968
2. 1969 – 1973
3. 1974 – 1978
4. 1979 – 1983
5. 1984 – 1988
6. 1989 – 1993
7. 1994 – 1998
8. 1999 – 2003
9. 2004 – 2008
10. 2009 – 2013
11. 2014 – 2018

Select the statement that best describes your level of experience of supervising occupational therapy students?

1. **Novice** – Have not or new to student supervision; co-supervised and/or supported student placements.
2. **Advanced Beginner** – Primary supervisor for short 1-2 week placement.
3. **Competent** – Primary supervisor for 5-8 week student placements with moderate support from supervisors and colleagues.
4. **Proficient** – Supervises students of all years and complexities with minimal supports from colleagues. Supports other staff taking students.
5. **Expert** – Supervises students and provides supervision training and mentorship to other staff taking students.

Work Place Demographics:

Select the option that best describes your primary scope of practice?

If you hold multiple scopes of practice, select the one that you complete MOST of the time. If you are currently un-employed or on extended leave, reflect on you last scope of practice.

Scope of Practice

1. Rehabilitation
2. Neurological
3. Paediatrics
4. Disability
5. Mental Health
6. Aged Care
7. Occupational health
8. Hand Therapy
9. Driving Assessment
10. Other – Please specify

Principle work setting – select your main work environment

1. Solo private practice
2. Group private practice
3. Other private practice
4. Aboriginal health service
5. Domiciliary service
6. Drug and alcohol service
7. Health promotion service
8. Rehabilitation / physical development service
9. Other community health care service
10. Hospital (excluding out patient)
11. Outpatient service
12. Residential aged care facility
13. Disability service
14. Hospice
15. Other residential health care facility
16. Sport centre/clinic
17. Other commercial business service
18. Educational facility
19. Correctional service
20. Defence forces
21. Other government department or agency
22. Other

Work envt 2

1. Major City – most capitals, major urban area such as Newcastle, Geelong, Gold Coast
2. Inner Regional – Cities and towns such as Hobart, Launceston, Mackay, Tamworth, Yass, Ballarat, Bathurst
3. Outer Regional – Cities and towns such as Albany, Moree, Darwin, Cairns, Devonport
4. Remote – Cities and towns such as Alice Springs, Roma, Esperance
5. Very Remote – Towns such as Tennant Creek, Longreach, Coober Pedy

State of primary practice

1. ACT
2. NSW
3. NT
4. QLD
5. SA
6. VIC
7. WA

Have you ever used feedback from a consumer to assist with your assessment of a student completing practice education?

1. Yes
2. No - (open text box) Can you please briefly outline why not?

Select the best option that describes how you gathered consumer feedback to assist with student learning:

1. Consumer filled in anonymous feedback form
2. Supervisor filled in form to document feedback from the consumer
3. Supervisor gathered verbal feedback from the consumer
4. Student filled in documented feedback from the consumer
5. Student gathered verbal feedback from the consumer
6. Consumer volunteered verbal feedback
7. Other – Please describe

Select the best option that describes how you used the feedback:

1. Direct - Supervisor used specific consumer feedback in supervision or SPEF-R documents.
2. Indirect - Supervisor used the information provided by the consumer to guide the supervisor’s assessment and mentorship of the student.
3. Other: Please describe

Reflecting on your work setting and consumer area of practice; (all questions were open text box replies)

1. What are some challenges with consumers providing feedback to students for the consumer?
2. What are some challenges with consumers providing feedback to students for the student?
3. What are some challenges with consumers providing feedback to students for the practice educator / setting?
4. What are some risks with consumers providing feedback to students for the consumer?
5. What are some risks with consumers providing feedback to students for the student?
6. What are some risks with consumers providing feedback to students for the practice educator / setting?
7. What are some benefits of consumers providing feedback to students for the consumer?
8. What are some benefits of consumers providing feedback to students for the student?
9. What are some benefits of consumers providing feedback to students for the practice educator / setting?
10. If you were to involve a consumer in providing feedback for students, what are the key things you would need to enable this to occur?

**NOTE**: Modifications made following testing and feedback from participants.

Order of Questions

- Original order of questions started with risks, then benefits and then challenges.
- Feedback stated that participants felt the order made their thinking jump between constructive, positive and ‘negative’ thinking.
- Recommendation by the pilot team not to put benefits first as it might bias the participants thinking.
- Outcome: Final order of questions were; challenges, risk then benefits.

Question terminology

- Original questions asked participants to reflect on barriers and enablers.
- Feedback stated that the term barriers and enablers encouraged participants to focus more on physical and object elements and recourses.
- Recommendation and outcome: change terms to challenges and benefits.

Questionnaire design

- No formatting was used in the questions.
- Participants suggested highlighting key works within each statement to assist with drawing the participants attention to difference in each statement.
- Outcome: Key words were underlined.
- Short answer option was used in the Qualtrics survey.
- Participants found the screen generated by the short answer design did not allow them to see enough of their response and discouraged them to write long answers.
- Outcome: Changed output to essay response to enable multiple lines of text to be seen at once.
