# Supplementary material for: Australian occupational therapists' perspectives of consumers authentically contributing to student learning during practice placements: ‘It just makes sense!’ but ‘we need a process’
Source: Aust Occup Ther J. 2022 Nov 13;69(6):753–65. doi: 10.1111/1440-1630.12853 (PMC10098716; doi:10.1111/1440-1630.12853)
Supplement: Supplementary file 2 — Data S2. Supporting Information [file AOT-69-753-s002.docx]

**Sample of Reflective Thematic Analysis**

| **Overall Theme** | **Candidate Themes** | **Codes** | **Additional Supporting Quotes** |
| --- | --- | --- | --- |
| **Personal capability of consumers and students will enable, inhibit and be developed by engaging in a feedback process** | Consumers are not capable  Consumers are not willing  Lack of confidence and trust in consumers ability to give constructive and safe feedback  Lack of confidence in student ability to receive, affectively, appropriate and accurately process and apply feedback  Consumer feedback could lead to inflated inaccurate confidence | Consumers not knowing what | “That the consumers feedback is very negative rather than constructive and upsets the student, decreases students confidence to function” (R36)  “It would require selection of patients that are self-reflective and well enough to have given such an indirect idea some of their attention” (R16)​  “Consumer doesn’t know what a good assessment or intervention should look like so doesn’t know what was incorrect about the service they received” (R24)  *“Students having difficulty accepting feedback objectively since they are often already somewhat sensitive with new experiences. Difficulty differentiating the reliability/objectivity of the consumer informant in the feedback” (R7)*  “They may not have training in how to provide appropriate feedback” (R78)  “They [the student] don't know how to contextualise or filter it - to glean the useful stuff, and the staff not to take to heart because it's not about you" (R8)  “Students often do not know how to phrase questions for feedback and if they do not have clear goals this is often an issue with obtaining appropriate feedback” (R66)  “Students often do not ask specific feedback from the consumer and students can become defensive or upset if the feedback is not positive.” (R66)  “It allows the opportunity for students to learn how to receive and deal with feedback in a professional and timely manner which is all part of the job as an occupational therapist.” (R38)  “Genuine and meaningful feedback from the people they are working with.  Will really challenge them to improve their skills.  Will set the tone for clinical practice where they need to be client-centred.  Will teach students that we need to seek feedback from the consumers we are working with.  Get students comfortable with consumers being in a more powerful role” (R12)  *“Can be very powerful for student particularly if positive” (R40)* |
|  |  | Consumers not knowing why |  |
|  |  | Consumer willingness |  |
|  |  | Impact on therapy |  |
|  |  | Consumers not able |  |
|  |  | Consumer fearful of giving negative feedback |  |
|  |  | Impact on therapy |  |
|  |  | Student ability to process |  |
|  |  | Student ability to filter |  |
|  |  | Student ability to learn |  |
|  |  | Student ability to apply |  |
|  |  | Consumer feedback can be too confronting |  |
|  | Consumer are empowered  Consumer is heard and valued | Improved consumer safety |  |
|  |  | Improved consumer empowerment |  |
|  |  | Improved consumer value |  |
|  |  | Improved consumer engagement |  |
|  | Powerful student development  Consumer feedback enable authentic learning from real world experiences | Increase student confidence |  |
|  |  | Student client centered practice |  |
|  |  | Develop student communication |  |
|  |  | Authentic learning |  |
|  |  | Real life direct |  |
| **An educator-controlled process to ensure safety for all stakeholders is required for time poor practice contexts** | Educators didn’t consider  Current assessment and processes do not support consumers giving feedback | Current assessment structure does not support | “Formal surveys or questionnaires would have to be established to allow for this also, pathways in place to request that feedback is provided rather than just accepting the feedback that is provided voluntary.” (R37)  “Clear structure, tool / mechanism, support and guidance from uni about how to weight that feedback.” (R76)  “Consumer could end up feeling "responsible" for something they should not have to worry about”  “Protecting the welfare of the consumer” (R2)  “Time to vet and brief the consumer” (R23)  “I could also help the students to gather that feedback towards the end of their care for a client so that the client can reflect on the whole process rather than simply one session.” (R16)  *“Feedback can be considered by the supervisor and could be adjusted as required to be more constructive” (R25)*  *“Potentially a university form that would legitimise the process? Or an addition to the SPEF-R?” (R1)*  “*It varies and I am selective of the people [consumers] I would ask” (R63)*  *“It would take more time to get feedback from consumers – potentially taking away potential learning opportunities” (R13)* |
|  |  | Lack of awareness |  |
|  |  | Time constraints |  |
|  | Educator filtered consumer feedback is occurring at an individual level | Observations of consumers to responses to students |  |
|  |  | Consumer feedback on student led services |  |
|  |  | Directly from consumers |  |
|  | Clear safe processes are required that work within pressured practice context | Educates & prepares student |  |
|  |  | Educates & prepares consumer |  |
|  |  | Practice context specific |  |
|  |  | Service design considerations |  |
|  |  | Time efficient process |  |
|  |  | Criteria for consumer |  |
|  |  | Feedback tool: specific v open |  |
|  |  | Values consumer contribution |  |
|  |  | Improved organizational reputation and safety |  |
| **Us versus them: shifting control to consumers can disempower practice educators** |  | Power change | *"That the student is following a protocol that isn't particularly consumer/person-centred. This provides a conflict for the educator as the student is doing what they've learnt but it isn't supportive of the consumer's goals or the way they wish to spend their time." (R42)*  *“I don’t see this as a risk because I see this as I think it’s reflective of the real world, but students can witness consumers and educators disagree on priorities and a focus for care.” (R6)*  *“Student may be unduly focused on how patient is 'assessing' them, and therefore be distracted from the purpose of their assessment/session” (R74)*  *“Consumer feedback could give more weight to feedback that is provided – when given from client perspective enhance quality of student placements” (R29)*  *“Real collaboration, less power discrepancies.” (R39)*  *“Feedback provided by consumer may be positive while feedback from supervisor may be negative, students who lack insight around their own performance and the limitations of obtaining consumer feedback may then use this to justify their view of their performance” (R7)*  *“…consumers are with us because they need help, not to do a job giving feedback” (R60)* |
|  |  | Alternative perspectives |  |
|  |  | Conflicting feedback |  |
|  |  | Discrepancies |  |
|  |  | Educator supporting |  |
|  |  | Usefulness & Appropriateness |  |
|  |  | Inaccurate |  |
|  |  | Value of consumer voice |  |
